# Predicting Appropriate Hospital Admission of Emergency Department Patients with Bronchiolitis: Secondary Analysis

**DOI:** 10.2196/12591

**Published:** 2019-01-22

**Authors:** Gang Luo, Bryan L Stone, Flory L Nkoy, Shan He, Michael D Johnson

**Affiliations:** 1 Department of Biomedical Informatics and Medical Education University of Washington Seattle, WA United States; 2 Department of Pediatrics University of Utah Salt Lake City, UT United States; 3 Care Transformation Intermountain Healthcare Salt Lake City, UT United States

**Keywords:** bronchiolitis, appropriate hospital admission, emergency department, predictive model, machine learning

## Abstract

**Background:**

In children below the age of 2 years, bronchiolitis is the most common reason for hospitalization. Each year in the United States, bronchiolitis causes 287,000 emergency department visits, 32%-40% of which result in hospitalization. Due to a lack of evidence and objective criteria for managing bronchiolitis, clinicians often make emergency department disposition decisions on hospitalization or discharge to home subjectively, leading to large practice variation. Our recent study provided the first operational definition of appropriate hospital admission for emergency department patients with bronchiolitis and showed that 6.08% of emergency department disposition decisions for bronchiolitis were inappropriate. An accurate model for predicting appropriate hospital admission can guide emergency department disposition decisions for bronchiolitis and improve outcomes, but has not been developed thus far.

**Objective:**

The objective of this study was to develop a reasonably accurate model for predicting appropriate hospital admission.

**Methods:**

Using Intermountain Healthcare data from 2011-2014, we developed the first machine learning classification model to predict appropriate hospital admission for emergency department patients with bronchiolitis.

**Results:**

Our model achieved an accuracy of 90.66% (3242/3576, 95% CI: 89.68-91.64), a sensitivity of 92.09% (1083/1176, 95% CI: 90.33-93.56), a specificity of 89.96% (2159/2400, 95% CI: 88.69-91.17), and an area under the receiver operating characteristic curve of 0.960 (95% CI: 0.954-0.966). We identified possible improvements to the model to guide future research on this topic.

**Conclusions:**

Our model has good accuracy for predicting appropriate hospital admission for emergency department patients with bronchiolitis. With further improvement, our model could serve as a foundation for building decision-support tools to guide disposition decisions for children with bronchiolitis presenting to emergency departments.

**International Registered Report Identifier (IRRID):**

RR2-10.2196/resprot.5155

## Introduction

Bronchiolitis refers to inflammation of the bronchioles, the smallest air passages in the lungs, mainly seen in children below the age of 2 years [1]. More than one-third of children in the United States have been diagnosed with bronchiolitis by the age of 2 years [1]. In children below the age of 2 years, bronchiolitis is responsible for 16% of the hospitalizations and is the most common reason for hospitalization [2-5]. In the United States, bronchiolitis annually leads to approximately 287,000 emergency department (ED) visits [6], 128,000 hospitalizations [2], and US $1.73 billion in total inpatient costs (2009) [2].

**Figure 1:**
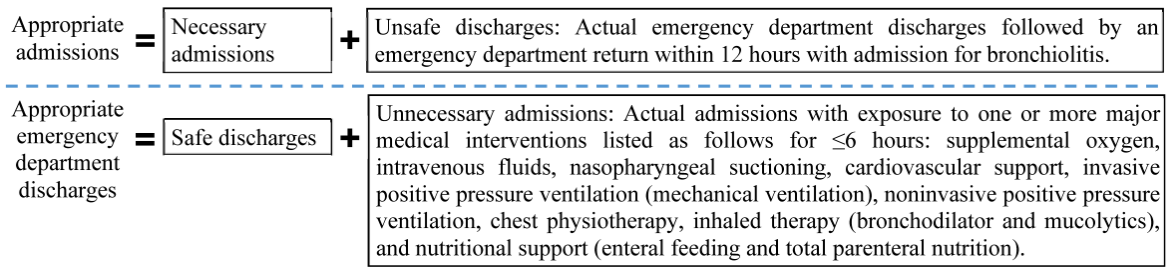
The operational definition of appropriate hospital admission for emergency department patients with bronchiolitis.

About 32%-40% of ED visits for bronchiolitis result in hospitalization [7-9]. Current clinical guidelines for bronchiolitis [10,11] acknowledge that due to a lack of evidence and objective criteria for managing bronchiolitis, clinicians often make ED disposition decisions of hospitalization or discharge to home subjectively [4,12]. This uncertainty in bronchiolitis management leads to large practice variation [3,12-23], increased iatrogenic risk, suboptimal outcomes, and wasted healthcare resources resulting from unnecessary admissions and unsafe discharges [15,21,24]. Approximately 10% of infants with bronchiolitis experience adverse events during hospital stay [25]. By examining the distributions of multiple relevant attributes of ED visits for bronchiolitis and using a data-driven method to determine two threshold values, we recently developed the first operational definition of appropriate hospital admission for ED patients with bronchiolitis [26]. Appropriate admissions cover both necessary admissions (actual admissions that are necessary) and unsafe discharges (Figure 1). Appropriate ED discharges cover both safe discharges and unnecessary admissions. Unsafe discharges are defined based on early ED returns. Unnecessary admissions are defined based on brief exposure to certain major medical interventions (Figure 1). Brief exposure was defined as exposure of ≤6 hours, with the threshold value of 6 hours chosen conservatively based on the median duration of major medical interventions received by a subset of patients who tended to have been admitted unnecessarily. Based on the operational definition, we showed that 6.08% of ED disposition decisions for bronchiolitis were inappropriate [26].

Thus far, several models have been built for predicting hospital admission in ED patients with bronchiolitis [7-9,27-29]. As our review paper [30] pointed out, these models have low accuracy and incorrectly assume that actual ED disposition decisions are always appropriate. An accurate model for predicting appropriate hospital admission can guide ED disposition decisions for bronchiolitis and improve outcomes. This model, which is yet to be built, would be particularly useful for less experienced clinicians, including junior clinicians and those in general practice who attend to children infrequently [31]. The objective of this study was to build the first model to predict appropriate hospital admission for ED patients with bronchiolitis. The dependent variable of the appropriate ED disposition decision is categorical and has two possible values: appropriate admission and appropriate ED discharge. Accordingly, the model uses clinical and administrative data to conduct binary classification.

## Methods

### Study Design and Ethical Approval

In this study, we performed secondary analysis of retrospective data. The Institutional Review Boards of the University of Washington Medicine, University of Utah, and Intermountain Healthcare reviewed and approved this study and waived the need for informed consent for all patients.

### Patient Population

Our patient cohort consisted of children below the age of 2 years who visited the ED for bronchiolitis in 2013-2014 at any of the 22 Intermountain Healthcare hospitals. Intermountain Healthcare is the largest healthcare system in Utah, with 22 hospitals and 185 clinics delivering ~85% of pediatric care in Utah [32]. Similar to our previous paper [26], we adopted the approach used in Flaherman et al [33-35] to identify as many ED visits for bronchiolitis as possible. This approach included patients with an ED or hospital International Classification of Diseases, Ninth Revision, Clinical Modification (ICD-9-CM) primary discharge diagnosis code of bronchiolitis or bronchitis (466.x), viral pneumonia (480.x), adenoviral infection (079.0), rhinovirus infection (079.3), respiratory infection due to influenza (487.0 or 487.1), respiratory syncytial virus (079.6), H1N1 influenza (488.1, 488.11, or 488.12), influenza due to identified avian influenza virus (488, 488.0, 488.01, or 488.02), or influenza due to novel influenza A (488.81 or 488.82). Any of these discharge diagnosis codes, rather than only the discharge diagnosis code of bronchiolitis, could be assigned to an ED visit for bronchiolitis. In addition, this approach included all patients with any of the abovementioned codes as a nonprimary diagnosis code, as long as the ICD-9-CM primary diagnosis code was any of the following: apnea (786.03), shortness of breath (786.05), tachypnea (786.06), wheezing (786.07), other respiratory abnormalities (786.09), cough (786.2), fever (780.60 or 780.61), acute nasopharyngitis (460), acute upper respiratory infections (465.x), other specified viral infection (079.89), urinary tract infection (599.0), pneumonia unspecified organism (486), unspecified viral infection (079.99), volume depletion (276.5x), or respiratory failure (518.81 or 518.82) [26]. The ED visits for bronchiolitis captured by this approach in 2013-2014 are the focus of our study.

### Data Set

From Intermountain Healthcare’s enterprise data warehouse, we extracted a clinical and administrative data set containing information of our patient cohort’s inpatient stays, ED visits, and outpatient visits at Intermountain Healthcare in 2011-2014. Our patient cohort included children below the age of 2 years who visited the Intermountain Healthcare ED for bronchiolitis in 2013-2014. By starting the data set in 2011, we ensured that for each ED visit by a target patient in 2013-2014, the data set included the patient’s complete prior medical history recorded within Intermountain Healthcare and necessary for computing features (also known as independent variables).

### Features

The 35 candidate patient features fall into two disjoint categories. *Category 1* includes all known predictors of hospital admission in ED patients with bronchiolitis, which were consistently recorded at Intermountain Healthcare facilities and available as structured attributes in our data set [30,31]. These 15 predictors are age in days, gender, heart rate, respiratory rate, peripheral capillary oxygen saturation (SpO_2_), temperature, coinfection, rhinovirus infection, enterovirus infection, history of bronchopulmonary dysplasia, history of eczema, prior intubation, prior hospitalization, prematurity, and dehydration. For any vital sign that was recorded more than once during the ED visit, we used its last value as its feature value. Among all recorded values, the last value most closely reflected the patient’s status at the time of ED disposition.

*Category 2* consists of 20 features suggested by our team’s clinical experts BLS, MDJ, and FLN: race, ethnicity, insurance category (public, private, or self-paid or charity), the ED visit’s acuity level (resuscitation, emergent, urgent, semiurgent, or nonurgent), chief complaint, number of consults during the ED visit, number of laboratory tests ordered during the ED visit, number of radiology studies ordered during the ED visit, number of X-rays ordered during the ED visit, length of ED stay in minutes, hour of ED disposition, whether the patient is up-to-date with his/her immunizations, diastolic blood pressure, systolic blood pressure, weight, wheezing (none, expiratory, inspiratory and expiratory, or diminished breath sounds), retractions (none, one location, two locations, or three or more locations), respiratory syncytial virus infection, language barrier to learning, and whether the patient has any other barrier to learning. For either attribute of wheezing and retractions that was recorded more than once during the ED visit, we used its last value as its feature value. Among all recorded values, the last value most closely reflected the patient’s status at ED disposition time.

Based on the timestamp, all candidate features were available as structured attributes in our data set before the time of ED disposition. We used these features to build predictive models.

### Data Analysis

#### Data Preparation

For each ED visit by a patient below the age of 2 years for bronchiolitis in 2013-2014, we used our previously developed operational definition of appropriate admission [26] (Figure 1) to compute the dependent variable’s value. For each numerical feature, we examined the data distribution, used the upper and lower bounds given by our team’s ED expert MDJ to identify invalid values, and replaced each invalid value with a null value. All temperatures<80°F or >110°F, all weights>50 pounds, all systolic blood pressure values of 0, all SpO_2_ values>100%, all respiratory rates>120 breaths/minute, and all heart rates<30 or >300 beats/minute were regarded as physiologically impossible and invalid. To ensure that all data were on the same scale, we standardized each numerical feature by first subtracting its mean and then dividing by its SD. We focused on 2 years of data for ED visits for bronchiolitis (2013-2014). Data from the first year (2013) were used to train predictive models. Data from the second year (2014) were used to evaluate model performance, reflecting use in practice.

#### Performance Metrics

As shown in Table 1 and the formulas below, we used six standard metrics to measure model performance: accuracy, sensitivity, specificity, positive predictive value (PPV), negative predictive value (NPV), and area under the receiver operating characteristic curve (AUC). For instance, false negative (FN) is the number of appropriate admissions that the model incorrectly predicts to be ED discharges. Sensitivity measures the proportion of appropriate admissions that the model identifies. Specificity measures the proportion of appropriate ED discharges that the model identifies.

Accuracy=(TP+TN)/(TP+TN+FP+FN)

Sensitivity=TP/(TP+FN)

Specificity=TN/(TN+FP)

PPV=TP/(TP+FP)

NPV=TN/(TN+FN)

TP is true positive, TN is true negative, and FP is false negative.

For the six performance metrics, we conducted 1000-fold bootstrap analysis [36] to compute their 95% CIs. On each bootstrap sample of the 2014 data, we computed our model’s performance metrics. For each of the six performance metrics, the 2.5th and 97.5th percentiles in the 1000 bootstrap samples specified its 95% CI.

**Table 1:** The error matrix.

Class	Appropriate admission	Appropriate emergency department discharge
Predicted admission	True positive	False positive
Predicted emergency department discharge	False negative	True negative

To show the sensitivity-specificity tradeoff, we plotted the receiver operating characteristic curve. The calibration of a model refers to how well the predicted probabilities of appropriate admission match with the fractions of appropriate admissions in subgroups of ED visits for bronchiolitis. To show model calibration, we drew a calibration plot [36]. A perfect calibration curve would coincide with the diagonal line. In addition, we used the Hosmer-Lemeshow goodness-of-fit test [36] to evaluate model calibration.

#### Classification Algorithms

We used Weka [37], a widely used open-source machine learning and data mining toolkit, to build machine learning classification models. Machine learning studies computer algorithms that learn from data, such as random forest, support vector machine, and neural network, and has won most data science competitions [38]. Weka integrates many commonly used machine learning algorithms and feature-selection techniques. We considered all 39 machine learning classification algorithms in the standard Weka package and adopted our previously developed automatic machine learning model selection method [39] and the training data of 2013 to automatically select the algorithm, feature-selection technique, and hyperparameter values among all the applicable ones. In a machine learning algorithm, hyperparameters are the parameters whose values are traditionally manually set by the machine learning software user before model training. An example of a hyperparameter is the number of decision trees used in a random forest classifier. Our automatic machine learning model selection method [39] uses the Bayesian optimization (also known as response surface) methodology to automatically explore numerous combinations of algorithm, feature-selection technique, and hyperparameter values and performs three-fold cross-validation to select the final combination maximizing the AUC. Compared to the other five performance metrics—accuracy, sensitivity, specificity, PPV, and NPV— AUC has the advantage of not relying on the cutoff threshold for deciding between predicted admission and predicted ED discharge.

### Demographic and Clinical Characteristics of the Patient Cohort

Tables 2 and 3 show the demographic and clinical characteristics of our patient cohort: children below the age of 2 years who visited the ED for bronchiolitis in 2013 and 2014, respectively. The characteristics are mostly similar between both years. About 40.78% (1640/4022) and 38.26% (1368/3576) of ED visits for bronchiolitis ended in hospitalization in 2013 and 2014, respectively. About 35.80% (1440/4022) and 32.89% (1176/3576) of ED visits for bronchiolitis were deemed to be appropriate hospital admissions in 2013 and 2014, respectively.

Based on the *χ*^2^ two-sample test, for the 2013 data, the ED visits discharged to home and those ending in hospitalization showed the same distribution for gender (*P*=.49) and different distributions for race (*P*<.001), ethnicity (*P*=.01), and insurance category (*P*<.001). For the 2014 data, the ED visits discharged to home and those ending in hospitalization showed the same distribution for gender (*P*=.94) and race (*P*=.61) and different distributions for ethnicity (*P*<.001) and insurance category (*P*<.001). Based on the Cochran-Armitage trend test [41], for both the 2013 and 2014 data, the ED visits discharged to home and those ending in hospitalization showed different distributions for age (*P*<.001).

**Table 2:** Demographic and clinical characteristics of children under the age of 2 years who visited the emergency department at Intermountain Healthcare hospitals for bronchiolitis in 2013.

Characteristic		Emergency department visits (N=4022), n (%)	Emergency department visits discharged to home (N=2382), n (%)	Emergency department visits ending in hospitalization (N=1640), n (%)
**Age**				
	<2 months	518 (12.88)	211 (8.86)	307 (18.72)
	2 to <12 months	2424 (60.27)	1498 (62.89)	926 (56.46)
	12 to 24 months	1080 (26.85)	673 (28.25)	407 (24.82)
**Gender**				
	Male	2369 (58.90)	1414 (59.36)	955 (58.23)
	Female	1653 (41.10)	968 (40.64)	685 (41.77)
**Race**				
	American Indian or Alaska native	51 (1.27)	26 (1.09)	25 (1.52)
	Asian	49 (1.22)	20 (0.84)	29 (1.77)
	Black or African American	124 (3.08)	78 (3.27)	46 (2.80)
	Native Hawaiian or other Pacific Islander	321 (7.98)	160 (6.72)	161 (9.82)
	White	2940 (73.10)	1784 (74.90)	1156 (70.49)
	Unknown or not reported	537 (13.35)	314 (13.18)	223 (13.60)
**Ethnicity**				
	Hispanic	1321 (32.84)	826 (34.68)	495 (30.18)
	Non-Hispanic	2687 (66.81)	1549 (65.03)	1138 (69.39)
	Unknown or not reported	14 (0.35)	7 (0.29)	7 (0.43)
**Insurance**				
	Private	2436 (60.57)	1338 (56.17)	1098 (66.95)
	Public	1422 (35.36)	933 (39.17)	489 (29.82)
	Self-paid or charity	164 (4.08)	111 (4.66)	53 (3.23)
Asthma		207 (5.15)	72 (3.02)	135 (8.23)
Chronic complex condition [40]		296 (7.36)	60 (2.52)	236 (14.39)

**Table 3:** Demographic and clinical characteristics of children under the age of 2 years who visited the emergency department at Intermountain Healthcare hospitals for bronchiolitis in 2014.

Characteristic		Emergency department visits (N=3576), n (%)	Emergency department visits discharged to home (N=2208), n (%)	Emergency department visits ending in hospitalization (N=1368), n (%)
**Age**				
	<2 months	454 (12.70)	186 (8.42)	268 (19.59)
	2 to <12 months	2079 (58.14)	1379 (62.45)	700 (51.17)
	12 to 24 months	1043 (29.17)	643 (29.12)	400 (29.24)
**Gender**				
	Male	2059 (57.58)	1273 (57.65)	786 (57.46)
	Female	1517 (42.42)	935 (42.35)	582 (42.54)
**Race**				
	American Indian or Alaska Native	47 (1.31)	31 (1.40)	16 (1.17)
	Asian	68 (1.90)	40 (1.81)	28 (2.05)
	Black or African American	104 (2.91)	70 (3.17)	34 (2.49)
	Native Hawaiian or other Pacific Islander	284 (7.94)	180 (8.15)	104 (7.60)
	White	2795 (78.16)	1708 (77.36)	1087 (79.46)
	Unknown or not reported	278 (7.77)	179 (8.11)	99 (7.24)
**Ethnicity**				
	Hispanic	1071 (29.95)	727 (32.93)	344 (25.15)
	Non-Hispanic	2484 (69.46)	1464 (66.30)	1020 (74.56)
	Unknown or not reported	21 (0.59)	17 (0.77)	4 (0.29)
**Insurance**				
	Private	2175 (60.82)	1241 (56.20)	934 (68.27)
	Public	1256 (35.12)	860 (38.95)	396 (28.95)
	Self-paid or charity	145 (4.05)	107 (4.85)	38 (2.78)
Asthma		210 (5.87)	67 (3.03)	143 (10.45)
Chronic complex condition [40]		252 (7.05)	43 (1.94)	209 (15.28)

## Results

Our automatic machine learning model selection method [39] chose the random forest classification algorithm. Random forest can naturally handle missing feature values. Our model was built using this algorithm and the 33 features shown in Table 4. These features are sorted in descending order of their importance values, which were automatically computed by the random forest algorithm in Weka based on average impurity decrease. In general, the features related to the patient’s history are ranked lower than those reflecting the patient’s status in the current ED visit. This intuitively makes medical sense. Two candidate patient features—ethnicity and the ED visit’s acuity level—were not used in our model because they did not increase the model’s accuracy.

**Figure 2:**
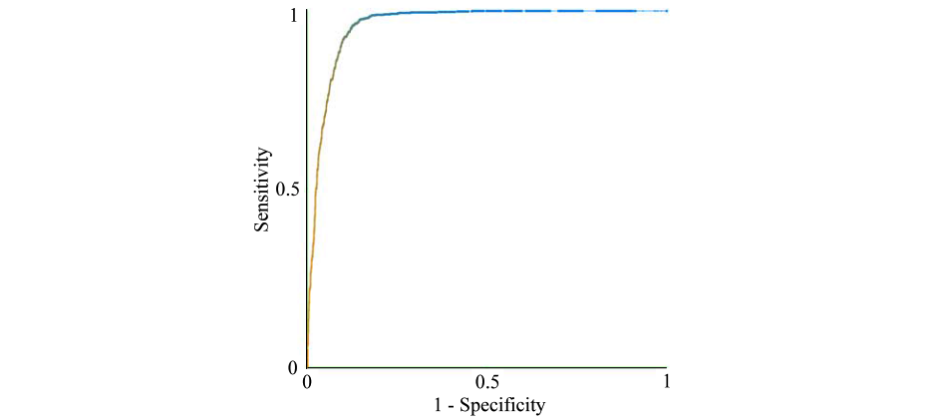
The receiver operating characteristic curve of our model.

Figure 2 shows the receiver operating characteristic curve of our model. Weka uses 50% as its default probability cutoff threshold for making binary classifications. Table 5 shows the error matrix of our model. Table 6 compares our model and the ED clinician’s disposition decision. Our model achieved an accuracy of 90.66% (3242/3576; 95% CI: 89.68-91.64), a sensitivity of 92.09% (1083/1176; 95% CI: 90.33-93.56), a specificity of 89.96% (2159/2400; 95% CI: 88.69-91.17), an AUC of 0.960 (95% CI: 0.954-0.966), a PPV of 81.80% (1083/1324; 95% CI: 79.67-83.80), and an NPV of 95.87% (2159/2252; 95% CI: 95.00-96.65). If we removed the insurance category feature, our model achieved a lower accuracy of 90.32% (3230/3576; 95% CI: 89.37-91.28), a lower sensitivity of 90.22% (1061/1176; 95% CI: 88.30-91.79), a specificity of 90.38% (2169/2400; 95% CI: 89.15-91.57), an AUC of 0.960 (95% CI: 0.955-0.966), a PPV of 82.12% (1061/1292; 95% CI: 79.94-84.15), and a lower NPV of 94.97% (2169/2284; 95% CI: 93.97-95.78). In comparison, the ED clinician’s disposition decision achieved an accuracy of 93.68% (3350/3576; 95% CI: 92.87-94.49), a sensitivity of 98.55% (1159/1176; 95% CI: 97.85-99.24), a specificity of 91.29% (2191/2400; 95% CI: 90.05-92.46), an AUC of 0.949 (95% CI: 0.942-0.956), a PPV of 84.72% (1159/1368; 95% CI: 82.83-86.69), and an NPV of 99.23% (2191/2208; 95% CI: 98.86-99.59).

**Table 4:** Features used in our model and their importance.

Feature	Importance based on average impurity decrease
Hour of ED^a^ disposition	0.42
Age in days	0.40
Whether the patient has any other barrier to learning	0.39
Length of ED stay in minutes	0.38
Number of laboratory tests ordered during the ED visit	0.37
Heart rate	0.37
Diastolic blood pressure	0.36
Gender	0.35
Temperature	0.35
Respiratory rate	0.34
Number of radiology studies ordered during the ED visit	0.34
Insurance category	0.34
Number of X-rays ordered during the ED visit	0.34
Systolic blood pressure	0.34
Weight	0.33
Chief complaint	0.32
SpO_2_^b^	0.32
Wheezing	0.32
Retractions	0.29
Number of consults during the ED visit	0.28
Whether the patient is up-to-date with his/her immunizations	0.27
Race	0.27
Enterovirus infection	0.25
Respiratory syncytial virus infection	0.24
Coinfection	0.24
Prior hospitalization	0.22
Prior intubation	0.22
Dehydration	0.20
Language barrier to learning	0.20
Rhinovirus infection	0.20
Prematurity	0.18
History of bronchopulmonary dysplasia	0.16
History of eczema	0.15

^a^ED: emergency department.

^b^SpO_2_: peripheral capillary oxygen saturation.

**Table 5:** The error matrix of our predictive model.

Class	Appropriate admission	Appropriate emergency department discharge
Predicted admission	1083	241
Predicted emergency department discharge	93	2159

**Table 6:** A comparison of our model and the emergency department clinician’s disposition decision.

	Accuracy (%)	Sensitivity (%)	Specificity (%)	AUC^a^	PPV^b^ (%)	NPV^c^ (%)
Our model	90.66	92.09	89.96	0.960	81.80	95.87
The emergency department clinician’s disposition decision	93.68	98.55	91.29	0.949	84.72	99.23

^a^AUC: area under the receiver operating characteristic curve.

^b^PPV: positive predictive value.

^c^NPV: negative predictive value.

**Figure 3:**
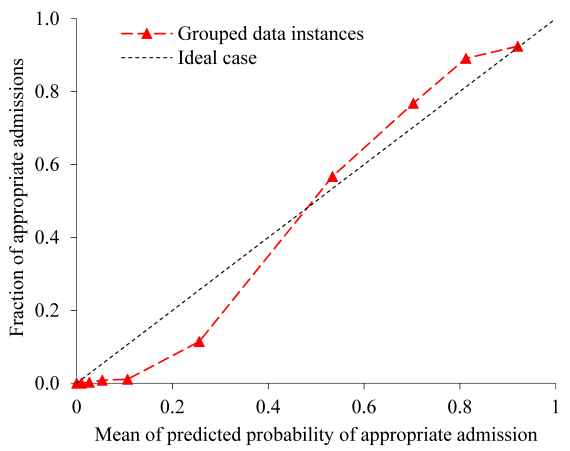
The calibration plot of our model by decile of predicted probability of appropriate admission.

Figure 3 shows the calibration plot of our model by decile of predicted probability of appropriate admission. The Hosmer-Lemeshow test showed imperfect calibration of the predicted probabilities and the actual outcomes (*P*<.001). When the predicted probability is <0.5, our model tends to overestimate the actual probability. When the predicted probability is >0.5, our model tends to underestimate the actual probability.

## Discussion

### Principal Results

**Figure 4:**
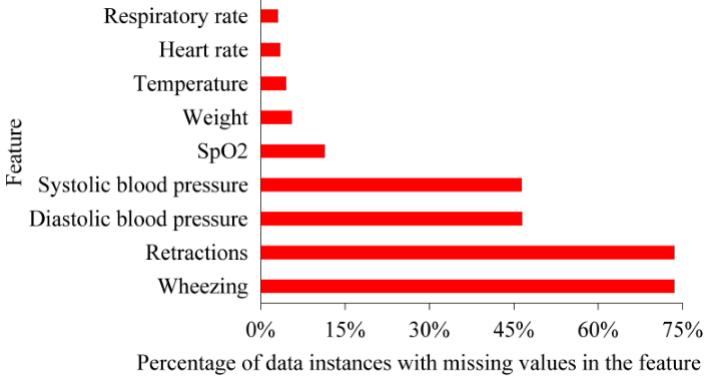
The degree of missing values of each feature with missing values. SpO_2_: peripheral capillary oxygen saturation.

**Figure 5:**
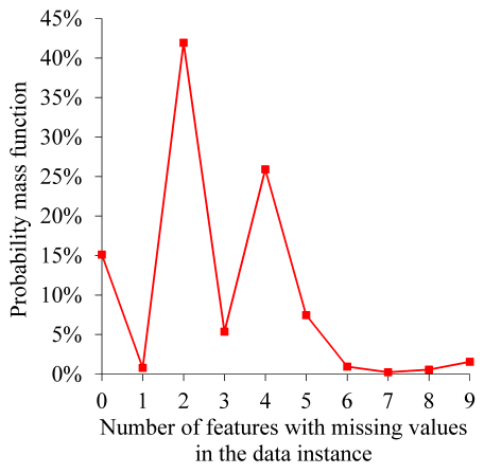
The probability mass function of the number of features with missing values in each data instance.

We developed the first machine learning classification model to accurately predict appropriate hospital admission for ED patients with bronchiolitis. Our model is a significant improvement over the previous models for predicting hospital admission in ED patients with bronchiolitis [7-9,27-29]. Our model has good accuracy, with five of the six performance metrics achieving a value ≥90% and the other metric achieving a value >80%. Although our model attained a 3.02% lower accuracy than Intermountain Healthcare clinicians’ ED disposition decisions (90.66% vs 93.68%), we still view our model as a step forward with great potential. Within 0.01 second, our model can output the prediction result for a new patient. With further improvement to boost its accuracy and automatically explain its prediction results [42,43], our model could be integrated into an electronic health record system and become the base of a decision-support tool to help make appropriate ED disposition decisions for bronchiolitis. At that time, a clinician could use the model’s output as a point of reference when considering the disposition decision. This could provide value, improve outcomes, and reduce health care costs for bronchiolitis, regardless of whether our future final model can achieve a higher accuracy than Intermountain Healthcare clinicians’ ED disposition decisions. Our faith in this model stems from the following considerations:

1. Intermountain Healthcare has several collaborative partnerships among its EDs and hospitals to facilitate coordination of pediatric specialty care and has completed multiple quality-improvement projects for bronchiolitis management. About 52.16% (3963/7598) of ED visits for bronchiolitis within Intermountain Healthcare occur at a tertiary pediatric hospital with an ED staffed by pediatric-specific clinicians. On average, the ED disposition decisions for bronchiolitis made at Intermountain Healthcare could be more accurate than those made at some other healthcare systems, especially those systems with general practice physicians or fewer pediatricians working in their EDs. Our model can be valuable for those systems, if it reaches a higher accuracy than the clinicians’ ED disposition decisions made at those systems. There is some evidence indicating this possibility. Most inappropriate ED disposition decisions are unnecessary admissions [26]. In our data set, 14.36% of hospital admissions from the ED were deemed unnecessary [26]. In the literature [44,45], this percentage is reported to be larger (20%-29%). To understand our model’s value for other systems, additional studies need to be conducted using data of those systems. This is an interesting area for future work.
2. Figure 4 shows the degree of missing values of each feature with missing values. Figure 5 shows the probability mass function of the number of features with missing values in each data instance. In our data set, several attributes have numerous missing values because those values were either recorded on paper or occasionally undocumented and therefore were not available in Intermountain Healthcare’s electronic health record system. In particular, wheezing and retractions values were missing for 73.56% (5589/7598) of ED visits for bronchiolitis. Systolic and diastolic blood pressure values were missing for 46.49% (3532/7598) of ED visits for bronchiolitis. This could lower the model’s accuracy. In the future, these attributes are expected to be recorded more completely in Intermountain Healthcare’s newly implemented Cerner-based electronic health record system. After retraining our model on more complete Intermountain Healthcare data from future years, we would expect its accuracy to increase. In addition, multiple other healthcare systems like Seattle Children’s Hospital have been using the Cerner electronic health record system to record these attributes relatively completely for many years. Our model could possibly achieve a higher accuracy if trained with data from those systems. Both of these areas are interesting for future work.
3. When making ED disposition decisions for bronchiolitis, clinicians often face some level of uncertainty and would prefer to obtain a second opinion by a reasonably accurate predictive model, particularly if some technique is used to automatically explain the model’s prediction results. For this purpose, we can use our prior method [42,43] to automatically provide rule-based explanations for any machine learning model’s classification results with no accuracy loss.

When reporting the performance metrics, we used the default cut-off threshold that Weka chose in order to decide between predicted admission and predicted ED discharge. Different health care systems could emphasize different performance metrics and provide divergent weights to FPs and FNs. As is the case with predictive modeling, in general, a health care system can always adjust the cut-off threshold based on the system’s preferences.

### Comparison With Prior Work

Previously, researchers constructed several models to predict hospital admission in ED patients with bronchiolitis [7-9,27-29]. Table 7 compared these previous models with our model. Compared to our model, which predicts the appropriate ED disposition decision, the previous models are less accurate and incorrectly assume that actual ED disposition decisions are always appropriate. Our model uses data from more patients, more predictive features, and a more sophisticated classification algorithm than the previous models. As is the case with predictive modeling, in general, all of these features help improve our model’s accuracy.

Some aspects of our findings are similar to those of previous studies. In our data set, 39.59% (3008/7598) of ED visits for bronchiolitis ended in hospitalization. This percentage is within the 32%-40% range of hospital admission rates on ED visits for bronchiolitis reported in the literature [7-9].

### Limitations

This study has several limitations. First, it used data from a single health care system, Intermountain Healthcare, and did not test the generalizability of the results. In the future, studies should validate our predictive models using data from other healthcare systems. We are reasonably confident in our results, as our study was conducted in a realistic setting for finding factors generalizable to other US healthcare systems. “Intermountain Healthcare is a large healthcare system with EDs at 22 heterogeneous hospitals spread over a large geographic area, ranging from community metropolitan and rural hospitals attended by general practitioners and family doctors with constrained pediatric resources to tertiary care children’s and general hospitals in urban areas attended by sub-specialists. Each hospital has a different patient population, geographic location, staff composition, scope of services, and cultural background” [26].

**Table 7:** A comparison of our model and several previous models for predicting emergency department disposition decisions for bronchiolitis.

Model	ED^a^ visits (n)	Method for building the model	Features included in the final model	Accuracy (%)	Sensitivity (%)	Specificity (%)	AUC^b^	PPV^c^ (%)	NPV^d^ (%)
Our model	7599	Random forest	As listed in the Results section	90.66	92.09	89.96	0.960	81.80	95.87
Walsh et al [27]	119	Neural network ensemble	Age, respiratory rate after initial treatment, heart rate before initial treatment, oxygen saturation before and after initial treatment, dehydration, maternal smoking, increased work of breathing, poor feeding, wheezes only without associated crackles, entry temperature, and presence of both crackles and wheezes	81	78	82	—^e^	68	89
Marlais et al [7]	449	Scoring system	Age, respiratory rate, heart rate, oxygen saturation, and duration of symptoms	—	74	77	0.81	67	83
Destino et al [28]	195	Single variable	The Children’s Hospital of Wisconsin respiratory score	—	65	65	0.68	—	—
Laham et al [8]	101	Logistic regression	Age, need for intravenous fluids, hypoxia, and nasal wash lactate dehydrogenase concentration	80	81	77	0.87	88	66
Corneli et al [9]	598	Decision tree	Oxygen saturation, the Respiratory Distress Assessment Instrument score computed from wheezing and retractions, and respiratory rate	—	56	74	—	—	—
Walsh et al [29]	300	Logistic regression	Age, dehydration, increased work of breathing, and heart rate	—	91	83	—	62	—

^a^ED: emergency department

^b^AUC: area under the receiver operating characteristic curve

^c^PPV: positive predictive value

^d^NPV: negative predictive value

^e^The performance metric is unreported in the original paper describing the model.

Second, despite being an integrated healthcare system, Intermountain Healthcare does not have complete clinical and administrative data on all of its patients. Our data set missed information on patients’ health care use that occurred at non-Intermountain Healthcare facilities. Inclusion of data from those facilities may lead to different results, but we do not expect this inclusion to significantly change our results. Intermountain Healthcare delivers ~85% of pediatric care in Utah [32]. Hence, our data set is reasonably complete with regard to capturing health care use among bronchiolitis patients in Utah.

Third, our operational definition of appropriate hospital admission is imperfect and excludes factors such as availability of patient transportation, preference of the patient’s parents, and hour of ED disposition [26]. Many of these factors are often undocumented in patient records. For some hospital admissions from the ED that were regarded as unnecessary based on our operational definition, the original admission decisions could be made because of these factors.

Finally, besides the features used in the study, other features could help improve the model’s accuracy. Finding new predictive features is an interesting area for future work.

### Conclusions

Our model can predict appropriate hospital admission for ED patients with bronchiolitis with good accuracy. In particular, our model achieved an AUC of 0.960. An AUC≥0.9 is considered outstanding discrimination [46]. With further improvement, our model could be integrated into an electronic health record system to provide personalized real-time decision support for making ED disposition decisions for bronchiolitis, which could help standardize care and improve outcomes for bronchiolitis.

## Abbreviations

AUC: area under the receiver operating characteristic curve
ED: emergency department
FN: false negative
FP: false positive
ICD-9-CM: International Classification of Diseases, Ninth Revision, Clinical Modification
NPV: negative predictive value
PPV: positive predictive value
SpO_2_: peripheral capillary oxygen saturation
TN: true negative
TP: true positive
